# Supplementary material for: Identification and Characterization of Antiviral Activity of Synthetic Compounds Against Mayaro Virus
Source: Pharmaceuticals (Basel). 2025 May 13;18(5):717. doi: 10.3390/ph18050717 (PMC12115251; doi:10.3390/ph18050717)
Supplement: Supplementary file 1 [file pharmaceuticals-18-00717-s001.zip › pharmaceuticals-3595122-supplementary.pdf]

Identification and Characterization of Antiviral Activity of Synthetic Compounds Against Mayaro Virus

Ana Paula Andreolla[1]; Andreia Cristine Koishi[1]; Alessandra Abel Borges[2]; Larissa Albuquerque de Oliveira[3], Viviane Guedes de Oliveira[3,4], Nerilson Marques Lima[3] , Eloah Pereira Ávila[3], Pedro Pôssa de Castro[3], Giovanni Wilson Amarante[3], Mauro Vieira de Almeida[3]; Juliano Bordignon[1]*; Claudia Nunes Duarte dos Santos[1]*

[1]Laboratório de Virologia Molecular, Instituto Carlos Chagas, ICC/Fiocruz, Rua Prof. Algacyr Munhoz Mader 3775, Cidade Industrial de Curitiba, Curitiba, Paraná, Brazil.

[2]Laboratório de Pesquisas em Virologia e Imunologia, Instituto de Ciências Biológicas e da Saúde (ICBS), Universidade Federal de Alagoas (UFAL), Av. Lourival Melo Mota, s/n, Tabuleiro do Martins, Maceió, Alagoas, Brazil.

[3]Departamento de Química, ICE, Universidade Federal de Juiz de Fora, Rua José Lourenço Kelmer s/n, Martelos, Juiz de Fora, Minas Gerais, Brazil.

[4]Instituto de Educação, Agricultura e Ambiente, IEAA, Universidade Federal do Amazonas, Rua 29 de agosto, Centro, Humaitá, Amazonas, Brazil.

*Corresponding authors: juliano.bordignon@fiocruz.br (J. Bordignon) and claudia.dossantos@fiocruz.br (C.N. Duarte dos Santos).

Supplementary Methodology

Standardization of anti-MAYV antiviral screening test

The susceptibility of various cell lines—C6/36, Vero E6, A549, Huh7.5, A172, SH-SY5Y, and THP-1-derived macrophages—to infection by MAYV-D was evaluated using IFI and the iHTS platform. Briefly, $1x10^4$ cells/well were seeded in 96-well plates and subsequently infected with MAYV_D at MOIs of 0.1, 1, and 10. Infected cultures were incubated for 24, 48, or 72 hours.

After each incubation period, the plates were fixed, stained, and analyzed using the IFA assay. Cell nuclei were stained with DAPI, while the percentage of MAYV_D infection was determined by staining with mAb 1G1, followed by an anti-mouse IgG antibody conjugated with Alexa Fluor 488.

For the THP-1 cell line, susceptibility to MAV-D infection was evaluated using a flow cytometry assay. THP-1 cells ($2x10^5$ cells/well) were seeded in 24-well plates and infected with MAYV_D at MOIs of 0.1, 1, and 10. The infection was performed for 24, 48, and 72 hours.

We selected the Huh7.5 cell line, a human hepatocarcinoma cell, for the screening of the compound library. To optimize the conditions for MAYV infection and the iHTS assay readout, we standardized the protocol using Huh7.5 cells. A total of $1x10^4$ and $2x10^4$ cells/well were seeded in 96-well plates and infected with different MOIs (0.02, 0.1, 0.5, 2.5, and 12.5) of MAYV, followed by incubation for 24 and 48 hours. After each incubation period, the plates were fixed, stained, and analyzed via IFA. Based on these evaluations, an MOI of 0.5 with a 24-hour incubation was determined to be the optimal condition for the IFA assay using iHTS. Cell concentration per well was validated through a statistical reliability test.

Based on the results obtained, three independent experiments were conducted to assess the statistical reliability and homogeneity of the percentage of viral infection across two concentrations of cells per well. The assay consisted of using previously seeded 96-well plates with either $1x10^4$ or $2x10^4$ cells/well and dividing them into MOCK (no infection, negative control) and MAYV (infected under optimized conditions, positive control) groups. The Z score was used to calculate the reliability value, using the following equation:

$$\boldsymbol{Z = ([(3 \times \sigma p) + (3 \times \sigma n)])/|\mu p - \mu n|}$$

Where σ is the standard deviation, and μ is the mean percentage of infection for the positive (p) and negative (n) wells.

Using the standardized infection conditions established for the screening tests, we determined the optimal concentrations of ribavirin and IFN-α 2A to serve as controls for evaluating *in vitro* anti-MAYV activity. Various concentrations of ribavirin (40–1.25 µM) and IFN-α 2A (4,000–31.25

IU/ml) were tested by incubating them with MAYV at an MOI of 0.5 for 24 hours. Following incubation, IFA was performed, and data on nuclear quantification and the percentage of infection were obtained.

Finally, using the standardized screening system, we evaluated two infection methods. In the first, the cells were infected, and the virus inoculum was removed after 1 hour (incubation/infection time). In the second, the virus inoculum was left in the culture throughout the entire experiment. The performance of these two methods was analyzed at different compound concentrations (200, 100, and 50 µM) using IFA. Nuclear quantitation and percentage of infection, both absolute and normalized, along with their standard deviations, were assessed to determine the most suitable method for the screening system.

Flow cytometry

After MAYV infection (24 or 48 hours), THP-1 cells were collected by centrifugation at 2500 rpm for 5 minutes and resuspended in 200 µL/well of blocking buffer (PBS + 5% FBS + 1% human serum) for 20 minutes at room temperature. Following incubation, the cells were centrifuged and fixed with 100 µL of Citofix/Citoperm (BD Biosciences, New Jersey, USA) for 20 minutes at room temperature. After another centrifugation, the cells underwent three washes with 200 µL/well of Perm/Wash solution (BD Biosciences), then incubated with the mAb 1G1 antibody (diluted 1:100 in Perm/Wash) for 30 minutes at 37 °C.

Subsequently, the cells were washed three times with Perm/Wash solution and incubated with an anti-mouse IgG antibody conjugated with Alexa Fluor 488 (diluted 1:400 in Perm/Wash) under the same conditions. Finally, the samples were washed three times with PBS and resuspended in 200 µL/well of PBS for immediate analysis by fluorescence cytometry. The quantification of infected cells was performed using a BD FACS Canto II cytometer (BD Biosciences), and the infection percentage data was analyzed with FlowJo software, version 10 (FlowJo LLC, San Fransciso, USA).

Virucidal assay

A mixture containing $2x10^5$ pfu/ml MAYV_D and the MNTC of each selected compound against MAYV (naringenin, LLA9A, chrysin, and ester C6) was incubated in microtubes for a final volume of 100 µl. After 1 hour, the virucidal activity of the compounds was assessed through a titration assay by plaque formation, as previously described (73). Briefly, the virus-compound mixture was diluted in a culture medium, and various dilutions of the mixture were inoculated into C6/36 cells.

Viral adsorption test

The ability of the selected anti-MAYV compounds (naringenin, LLA9A, chrysin, and ester C6) to inhibit viral adsorption was evaluated using 96-well plates previously seeded with $2x10^4$ Huh7.5 cells/well. MAYV_D (0.5 MOI) was inoculated along with the MNTC of each compound and incubated for 90 minutes at 4°C. After incubation, the cells were washed, and a warm culture medium was added. At 24 hpi and 37°C, the cells were fixed with cold methanol-acetone and analyzed by IFI. The supernatant was then subjected to virus titration by plaque assay.

Viral internalization test

The viral internalization assay consisted of infecting Huh7.5 cells previously seeded in 96-well plates with MAYV_D (0.5 MOI) for 90 minutes at 4°C. Following incubation, the MNTC of each selected anti-MAYV compound (naringenin, LLA9A, chrysin, and ester C6) was added, and the cells were incubated for an additional 90 minutes at 37°C. Following this second incubation, the cells were washed with PBS and treated with citrate buffer (40 mM citric acid, 10 mM potassium chloride, 135 mM sodium chloride, pH 3) for 1 minute. The cells were washed again, cultured with medium, and incubated for 24 hours at 37°C. After fixation with methanol-acetone, the cells were analyzed by IFI. The supernatant was then subjected to virus titration by plaque assay.

General procedure and characterization data for flavonoid derivatives

Naringenin derivatives

Procedure and characterization data for the derivatives of naringenin are described in the literature.[1]

Chrysin derivatives

Experimental data for ethers

The experimental data for 7*O*-hexyl-chrysin are described in literature by Cheng *et al* 2014. [2] For 7*O*-dodecyl-chrysin (Chrysin ether C12) and 7*O*-hexadecyl-chrysin (Chrysin ether C16), experimental procedures were carried out using Williamson ether synthesis.

To a solution of chrysin (1.0 mmol) in *N*,*N*-dimethylformamide (DMF) (5 mL) was added potassium *tert*-butoxide (1.1 mmol) and stirred at room temperature. Then, the *n*-alkyl bromide (1.1 mmol) and the reactional mixture was heated to 60°C and stirred for 24 h. After completion,

DMF was removed after extraction with water (50 mL) and dicloromethane (50 mL). The volatiles were evaporated under reduced pressure. The products were obtained after purification through chromatography column (elution ethyl acetate/hexane).

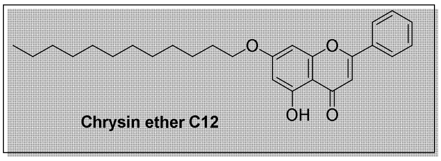

7-(dodecyloxy)-5-hydroxy-2-phenyl-4H-chromen-4-one (Chrysin ether C12): yellow solid (0.253 g, 72%). ^{1}H NMR (500 MHz, $CDCl_3$) δ 12.72 (s, 1H), 7.90 (d, *J* = 7.5 Hz, 2H), 7.60 – 7.50 (m, 3H), 6.68 (s, 1H), 6.51 (d, *J* = 2.6 Hz, 1H), 6.38 (d, *J* = 2.6 Hz, 1H), 4.11 – 4.01 (m, 2H), 1.83 (qui, *J* = 6.8 Hz, 2H), 1.48 (qui, *J* = 6.9 Hz, 2H), 1.43 – 1.26 (m, 16H), 0.90 (t, *J* = 6.9 Hz, 3H). ^{13}C NMR (126 MHz, $CDCl_3$) δ 182.5, 165.3, 163.9, 162.1, 157.8, 131.8, 131.8, 131.4, 129.1, 126.3, 126.2, 105.9, 105.8, 98.7, 93.2, 68.7, 31.9, 29.6, 29.6, 29.4, 29.0, 28.9, 28.62, 28.5, 26.0, 22.7, 14.1. Melting point: 88-90 °C.

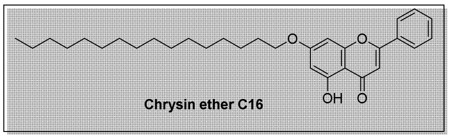

7-(hexadecyloxy)-5-hydroxy-2-phenyl-4H-chromen-4-one (Chrysin ether C16): yellow solid (0.253 g, 72%). ^{1}H NMR (500 MHz, $CDCl_3$) δ 12.72 (s, 1H), 7.94 – 7.88 (m, 2H), 7.60 – 7.50 (m, 3H), 6.68 (s, 1H), 6.51 (d, *J* = 2.2 Hz, 1H), 6.39 (d, *J* = 2.1 Hz, 1H), 4.05 (t, *J* = 6.6 Hz, 2H), 1.91 – 1.79 (m, 2H), 1.53 – 1.42 (m, 2H), 1.28 (s, 24H), 0.90 (t, *J* = 6.9 Hz, 3H). ^{13}C NMR (125 MHz, $CDCl_3$) δ 182.5, 165.3, 163.9, 162.1, 157.8, 131.8, 131.4, 129.1, 126.3, 105.9, 105.6, 98.6, 93.1, 68.7, 31.9, 29.7, 29.69, 29.67, 29.64, 29.60, 29.56, 29.51, 29.46, 29.38, 29.34, 29.0, 26.0, 14.1.

Experimental data for esters

The experimental data for 7*O*-acetyl-chrysin (Chrysin acetate) are described in the literature by Zhu *et al* 2014. [3] For 7*O*-hexanoyl-chrysin (Chrysin ester C6) and 7*O*-decanoyl-chrysin (Chrysin ester C10), experimental procedures were carried out using Steglich sterification.

To a solution of chrysin (1.1 mmol) in dichloromethane (DCM) was added the corresponding fatty acid (1.4 mmol), *N,N'*-Dicyclohexylcarbodiimide (DCC) (2.0 mmol), and catalytic dimethyl aminopyridine (DMAP) at 0 °C. The reaction mixture was stirred at room temperature for 24h. After completion, the solution was extracted with distilled water (10 mL). The volatiles were

evaporated under reduced pressure. The isolated products were obtained after purification through chromatography column (ethyl acetate/hexane).

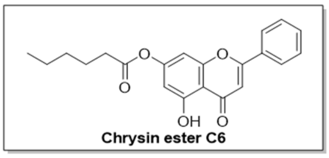

5-hydroxy-4-oxo-2-phenyl-4H-chromen-7-yl hexanoate (Chrysin ester C6): yellow solid (0.253 g, 72%). ^{1}H NMR (500 MHz, $CDCl_3$) δ 12.74 (s, 1H), 7.94 – 7.88 (m, 2H), 7.63 – 7.51 (m, 3H), 6.88 (d, J = 2.0 Hz, 1H), 6.75 (s, 1H), 6.59 (d, J = 2.0 Hz, 1H), 2.61 (t, J = 7.5 Hz, 2H), 1.80 (p, J = 7.4 Hz, 2H), 1.49 – 1.36 (m, 4H), 0.99 – 0.95 (m, 3H). ^{13}C NMR (126 MHz, $CDCl_3$) δ 182.9, 171.3, 164.7, 161.9, 156.8, 156.1, 132.2, 131.0, 129.2, 126.4, 108.9, 106.1, 105.5, 101.1, 34.4, 31.2, 24.5, 13.9.

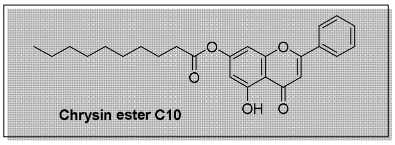

5-hydroxy-4-oxo-2-phenyl-4H-chromen-7-yl decanoate (Chrysin ester C10): yellow solid (0.330 g, 81%). ^{1}H NMR (500 MHz, $CDCl_3$) δ 4.80 (d, *J* = 5.7 Hz, 1H), 4.33 (d, *J* = 11.5 Hz, 1H), 4.20 (dd, *J* = 11.4, 6.4 Hz, 1H), 3.02 (s, 6H), 1.76 – 1.58 (m, 2H), 1.41 – 1.30 (m, 2H), 1.19 (s, 14H), 0.81 (t, *J* = 6.6 Hz, 4H). ^{13}C NMR (126 MHz, $CDCl_3$) δ 79.4, 69.7, 38.7, 37.7, 31.9, 31.1, 29.5, 29.46, 29.29, 29.26, 29.2, 24.8, 22.6, 14.1.

Carbamate-containing compounds

Procedure and characterization data for carbamate derivatives are described in the literature.[4,5]

Supplementary Figures

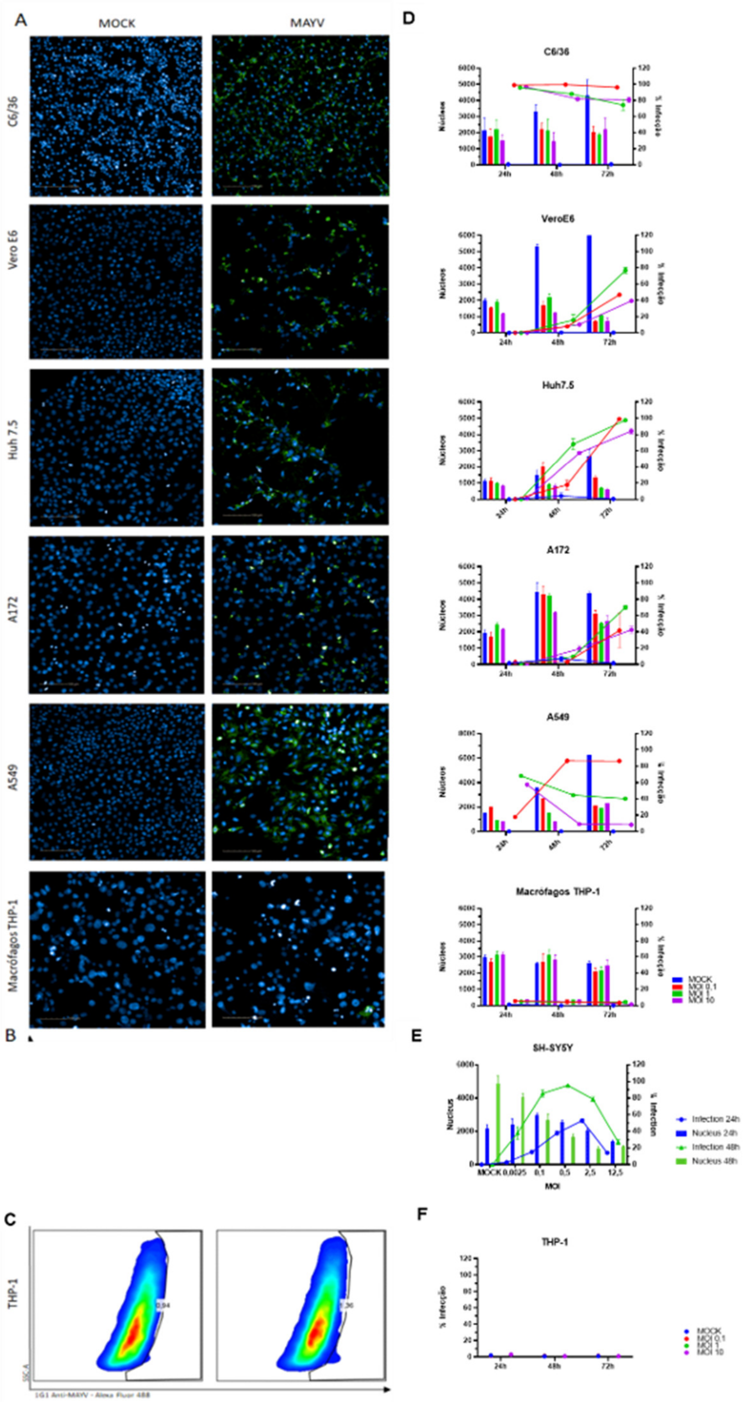

Supplementary Figure 1: Cell permittivity of different cell lines for MAYV_D. (A) The representative image of the permissiveness to MAYV_D (MOI 0.1) of cultures of C6/36, Vero E6, Huh-7.5, A172, A549 and THP-1 differentiated macrophages, by IFI after 48h of infection is shown. **(B)** Infection data and number of nuclei per well for each strain (except for THP-1) are shown for each MOI tested (0.1, 1 and 10) and time post infection (24, 48 and 72 h.p.i.). **(C)** THP-1 was evaluated by flow cytometry (MOI 0.1; 48h of infection) and **(D)** Infection data for THP-1 is shown for each MOI tested (1.1, 1 and 10) and time post infection (24, 48 and 72 h.p.i). Data from three biological replicates in technical triplicate are demonstrated. In blue, nuclei stained with DAPI and, in green, MAYV_D labeled with mAb1G1 followed by anti-mouse IgG antibody conjugated with Alexa Fluor 488. MOCK: uninfected cells after 48 h.p.i.; MAYV: cells infected with

MAYV MOI 0.1 and 48 h.p.i. Bars correspond to nuclear quantitation and lines correspond to the percentage of infected cells; blue: MOCK; Red: MOI 0.1; green: MOI 1; Purple: MOI 10. Bar in the MOCK C6/36 image corresponds to the reference value of 100 µm.

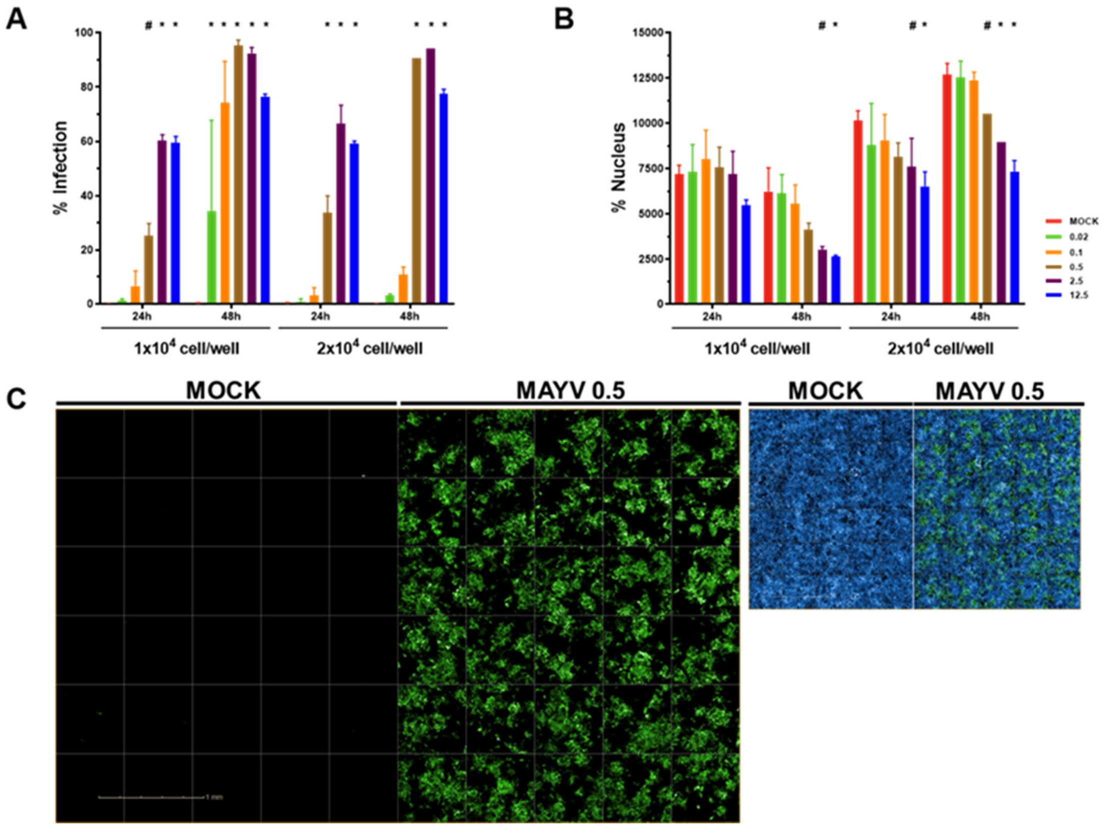

Supplementary Figure 2: Standardization of Huh-7.5 infection with MAYV_D for iHTS screening assay. Two different amounts of cells (1 and $2x10^4$ cells/well) were seeded and infected with MAYV_D by times (24 and 48 h.p.i.) and MOIs (12.5, 2.5, 0.5, 0.1 and 0.02) many different. The percentage of infected cells **(A)** and nuclear quantification **(B)** were analyzed by IFI. **(C)** The representative of the statistical reliability assay using half an uninfected 96-well plate (MOCK) and half an infected plate with standardized MAYV infection conditions ($2x10^4$ cells/well, MOI 0.5 per 24h). In bar charts A and B, in red MOCK, green MOI 0.02, orange MOI 0.1, brown MOI 0.5, purple MOI 2.5, blue MOI 12.5. In images (C), nuclear staining with DAPI in blue and MAYV staining in green using mAb 1G1 followed by anti-mouse IgG conjugated with Alexa Fluor 488. The bar corresponds to 1mm. The expressed data corresponds to mean values with the respective standard deviations of three independent experiments in technical triplicate. Significant difference represented by (*) when $p<0.001$ and (#) when $p<0.05$ when comparing the corresponding non-infected (MOCK) group, analyzing the data using ANOVA followed by Tukey's test, of three biological replicates in triplicate.

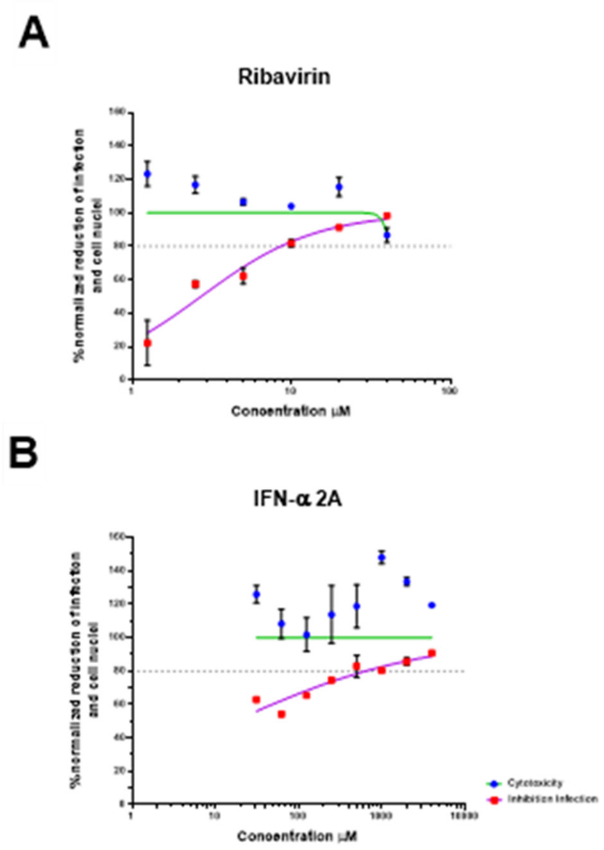

Supplementary Figure 3: Determination of use concentration of anti-MAYV activity controls. Two compounds, ribavirin **(A)** and IFN-α 2A **(B)** were serially diluted (1:2) in cell culture medium containing MAYV_D (MOI 0.5), incubated in Huh-7.5 cells and fixed 24 h.p.i. Data on the normalized percentage of nuclear quantification (blue circles with green line) and infection (red squares with purple line) are shown in a dose-response curve graph of three independent experiments in triplicate. Dotted line represents IC80.

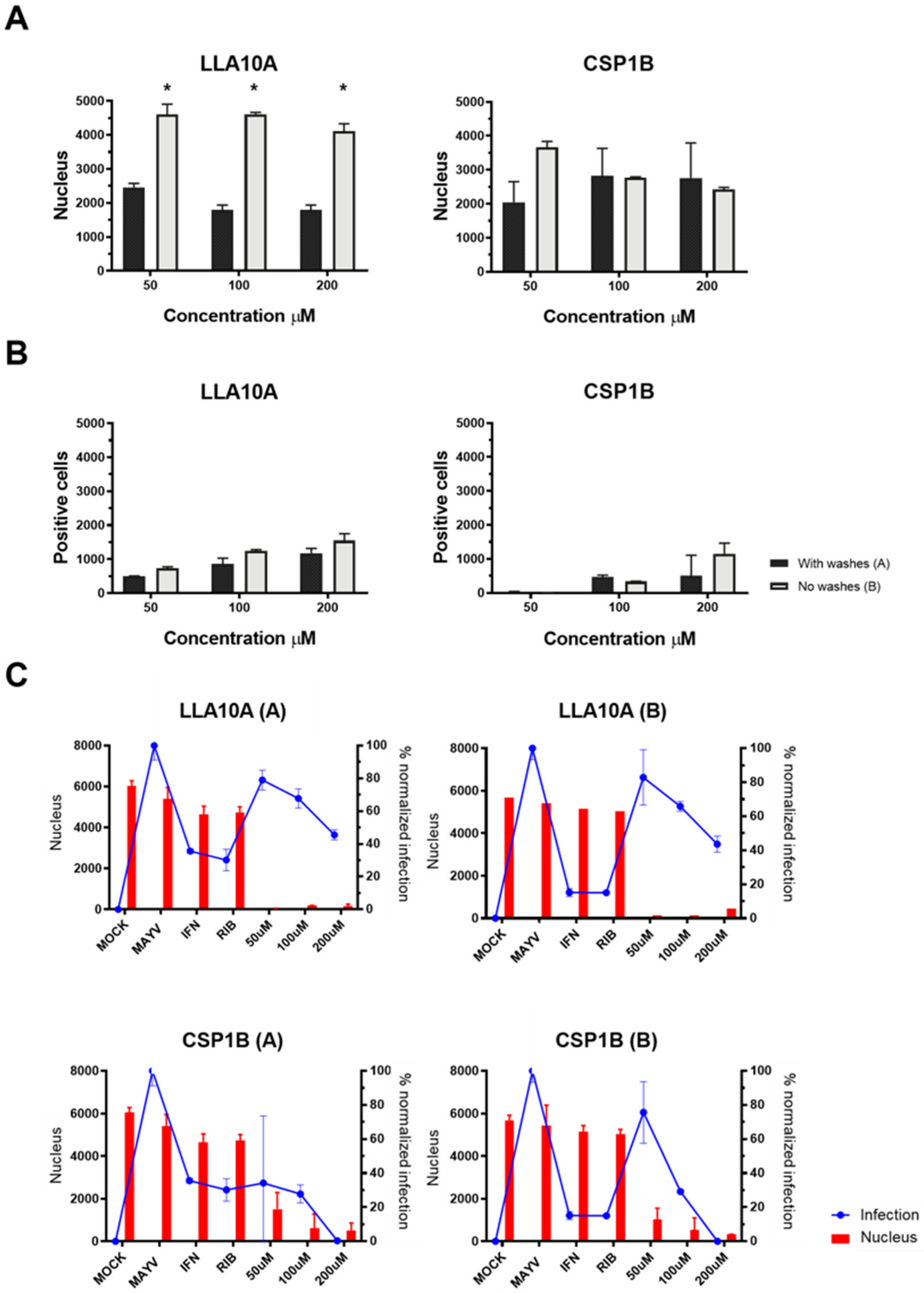

Supplementary Figure 4: Huh-7.5 infection methodologies with MAYV_D for compound screening in iHTS system. We used two methodologies in two different compounds, LLA10A and CSP1B and analyzed, after 24 hours of infection, the values: **(A)** absolute nuclei, **(B)** absolute number of positive cells infected with MAYV_D (black bars - methodology A ; gray bars – methodology B) and **(C)** the normalized data of the percentage of nuclear quantification and infection (bars and lines, respectively). The expressed data correspond to mean values with the respective standard deviations of three independent experiments in technical triplicate. Significant difference represented by (*) when p<0.001 when comparing the same concentration

in different methodologies, analyzing the data using two-way ANOVA followed by Tukey's test, three biological replicates in triplicate.

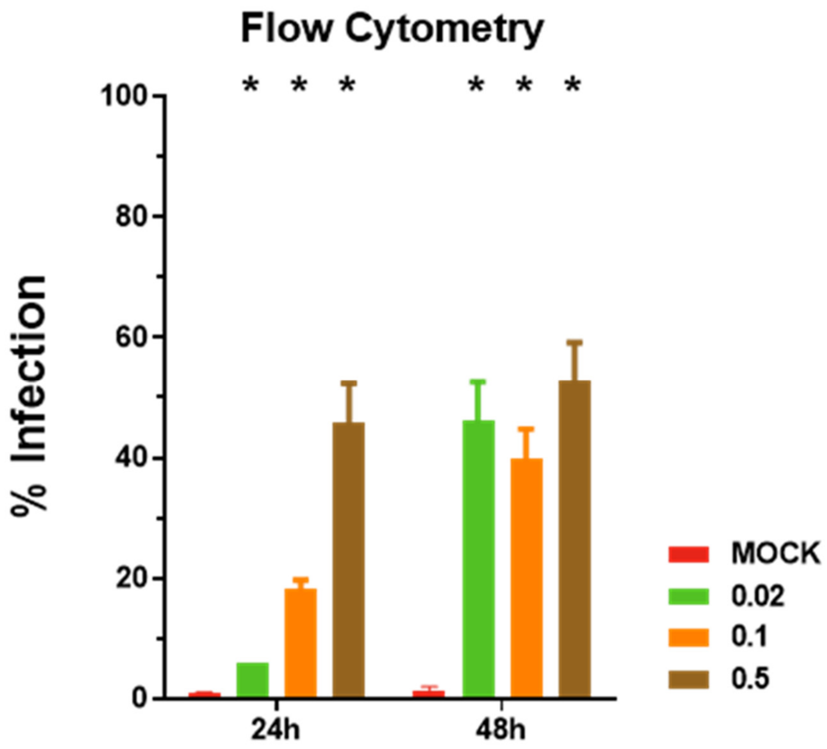

Supplementary Figure 5: Patterning Huh-7.5 infection with MAYV_D for flow cytometry. 2x105 cells/well were infected with different MOIs (0.02, 0.1 and 0.5) of MAYV and incubated for 24 and 48 h.p.i. Once fixed, labeled with mAb1G1 followed by anti-mouse IgG antibody conjugated with Alexa Fluor 488, data were acquired and the percentages of infection for each condition were analyzed. In red – MOCK, green – MOI 0.02, orange – MOI 0.1, brown – MOI 0.5. Mean data with their respective standard deviations from three independent experiments in technical triplicate. Significant difference represented by (*) when $p<0.001$ when comparing the corresponding uninfected control group (MOCK), analyzing the data using ANOVA followed by Tukey's test, from three biological replicates in triplicate.

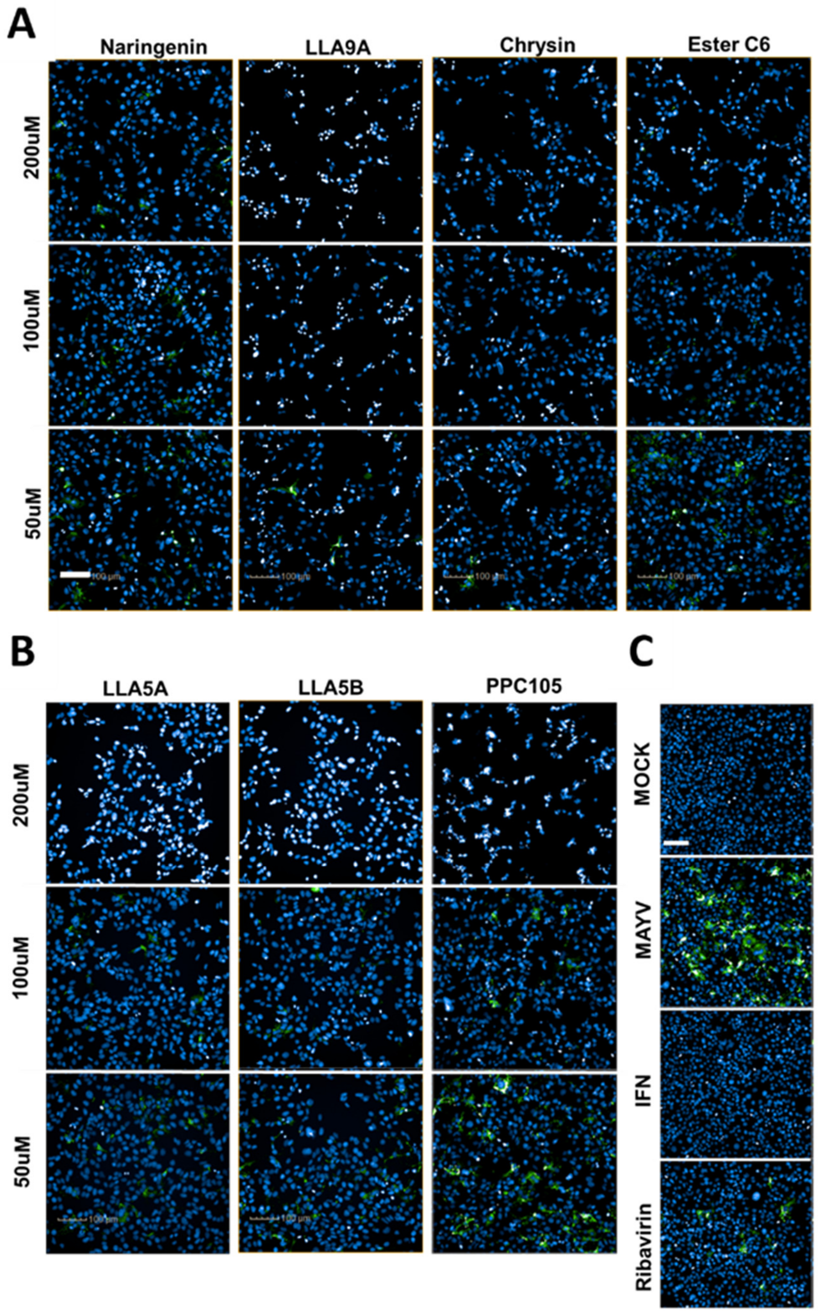

Supplementary Figure 6: IFA for iHTS. The representative of the images acquired by IFA of each tested concentration of the compounds that remained within the parameters are shown. **(A)** Compounds classified as anti-MAYV candidates; **(B)** Compounds discarded by SI or cytotoxicity; and **(C)** MOCK, MAYV, IFN and Ribavirin controls. In the images, the cell nucleus is marked in blue by DAPI and, in green, MAYV through mAb 1G1 followed by anti-mouse IgG antibody conjugated with Alexa Fluor 488. The white bars correspond to 100 μm.

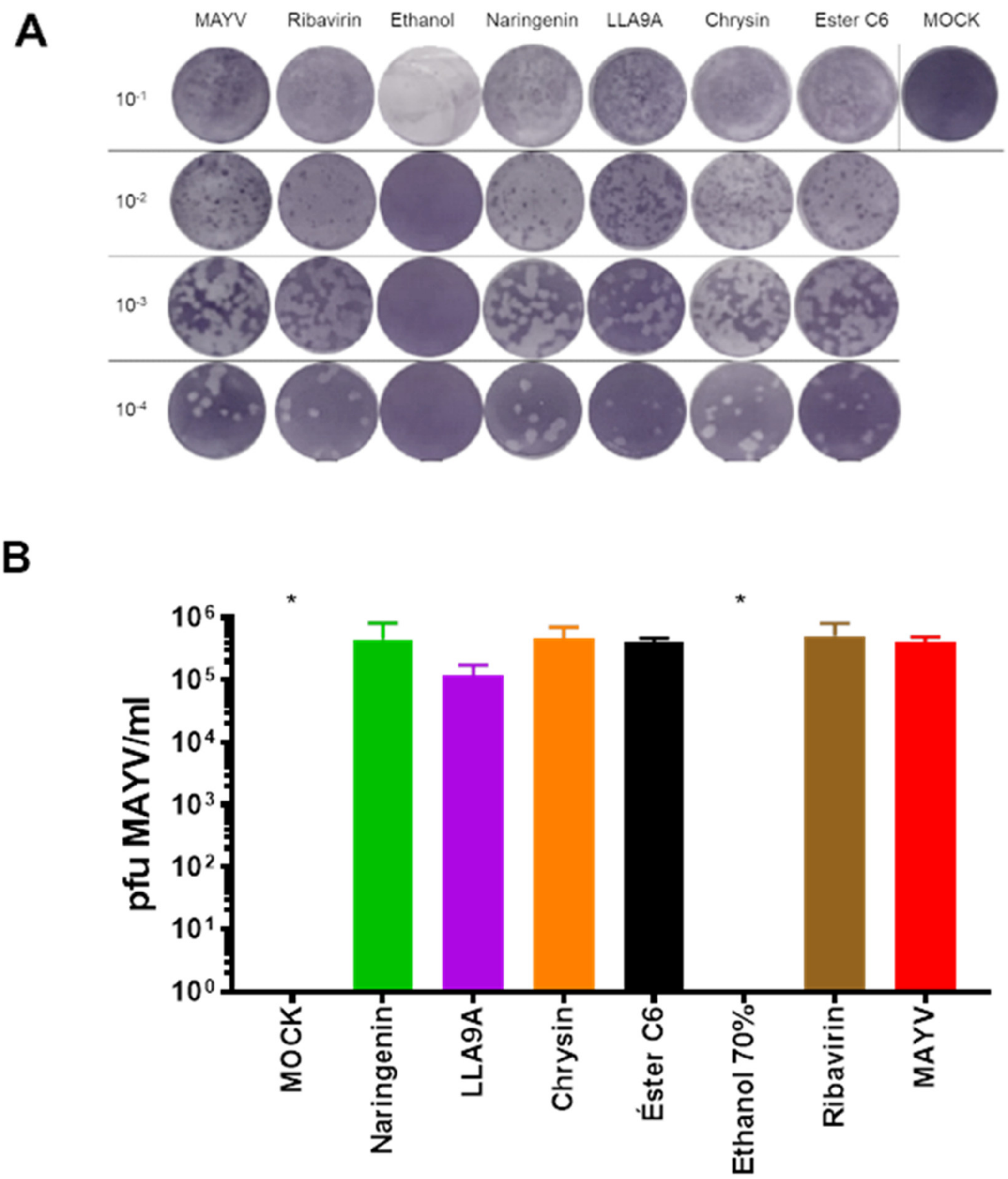

Supplementary Figure 7: Virucidal assay of candidate anti-MAYV compounds. MAYV_D was exposed to the DMNT of each candidate compound (naringenin - 200 µM, LLA9A – 50 µM, chrysin - 25 µM and C6 ester – 25 µM) in order to determine the virucidal action of the compound. **(A)** Titration by forming a plate stained with crystal violet and **(B)** the statistical analysis of the data obtained by counting the formed plates. Ethanol 70% was used as a virucidal control in the assay. Significant difference against the MAYV group is represented by (*) when compared using ANOVA followed by Tukey's test ($p<0.001$) of three biological replicates in triplicate.

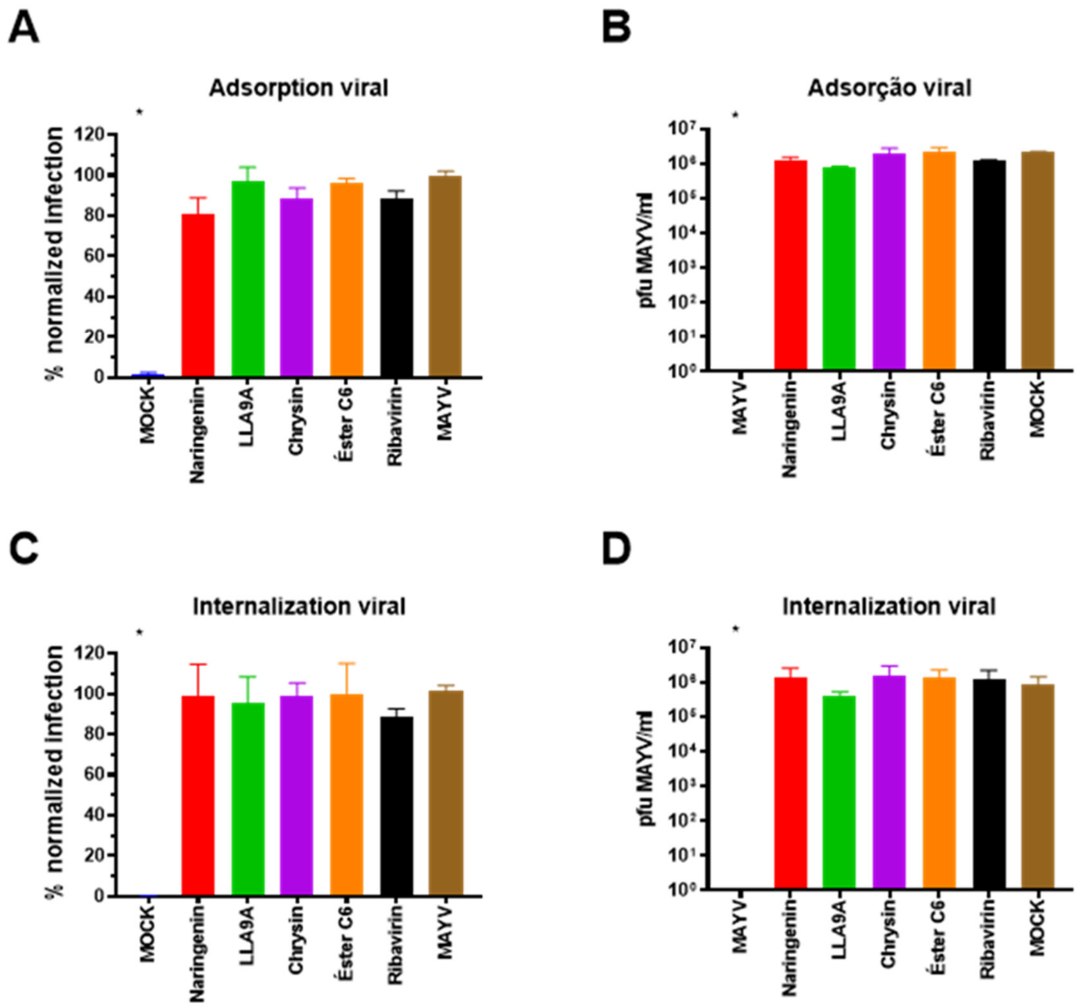

Supplementary Figure 8: **Adsorption and internalization viral assay of candidate anti-MAYV compounds.** The action of anti-MAYV compounds (naringenin - 200 µM, LLA9A – 50 µM, chrysin - 25 µM and C6 ester – 25 µM) were analyzed in terms of their ability to interact with MAYV_D at two different moments in replication, adsorption and internalization. **(A)** and **(C)** The analysis is demonstrated through the normalized data of the iHTS system, respectively, through the marking of mAb 1G1 followed by anti-mouse IgG antibody conjugated with Alexa Fluor 488. **(B)** and **(D)** quantification of viable viral particles from the supernatant from assays A and C, through viral titration assay by plaque formation, respectively. In both assays and techniques there was no statistical difference (*) in MAYV infection when analyzed using ANOVA followed by Tukey's test ($p<0.05$) of three biological replicates in triplicate.

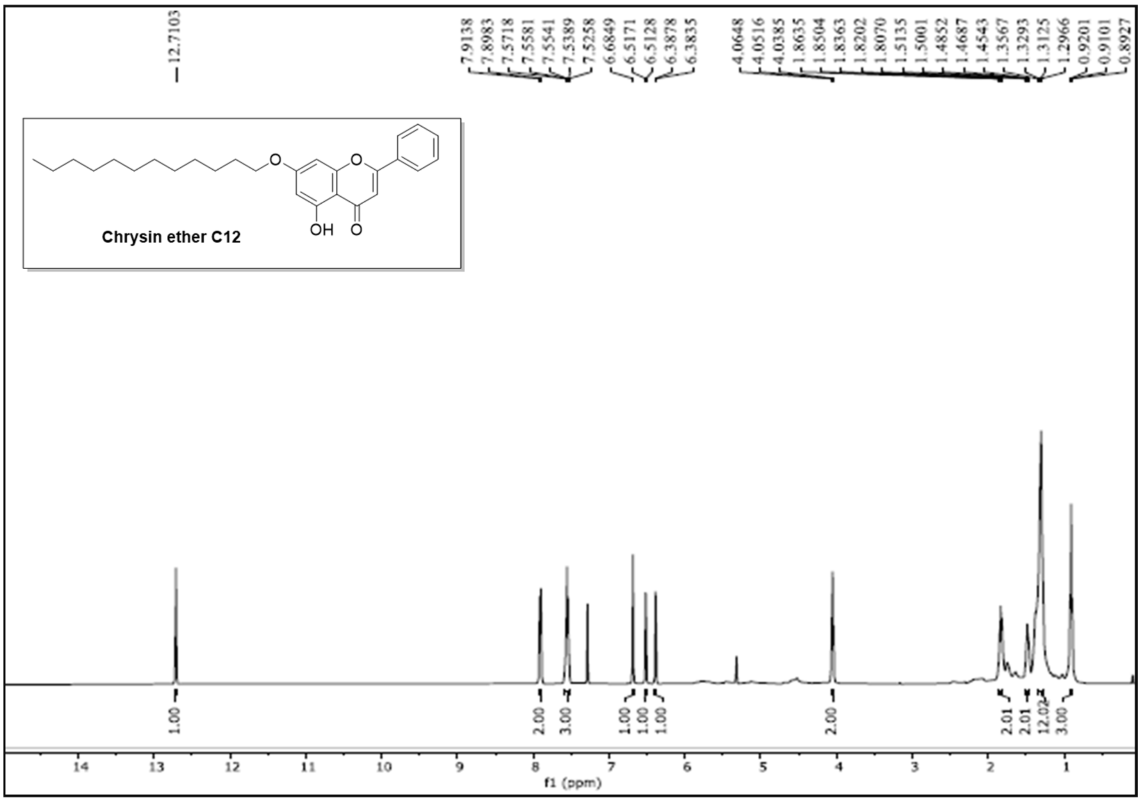

Supplementary Figure 9: ^{1}H NMR ($CDCl_3$, 500 MHz) spectrum of **Chrysin ether C12**.

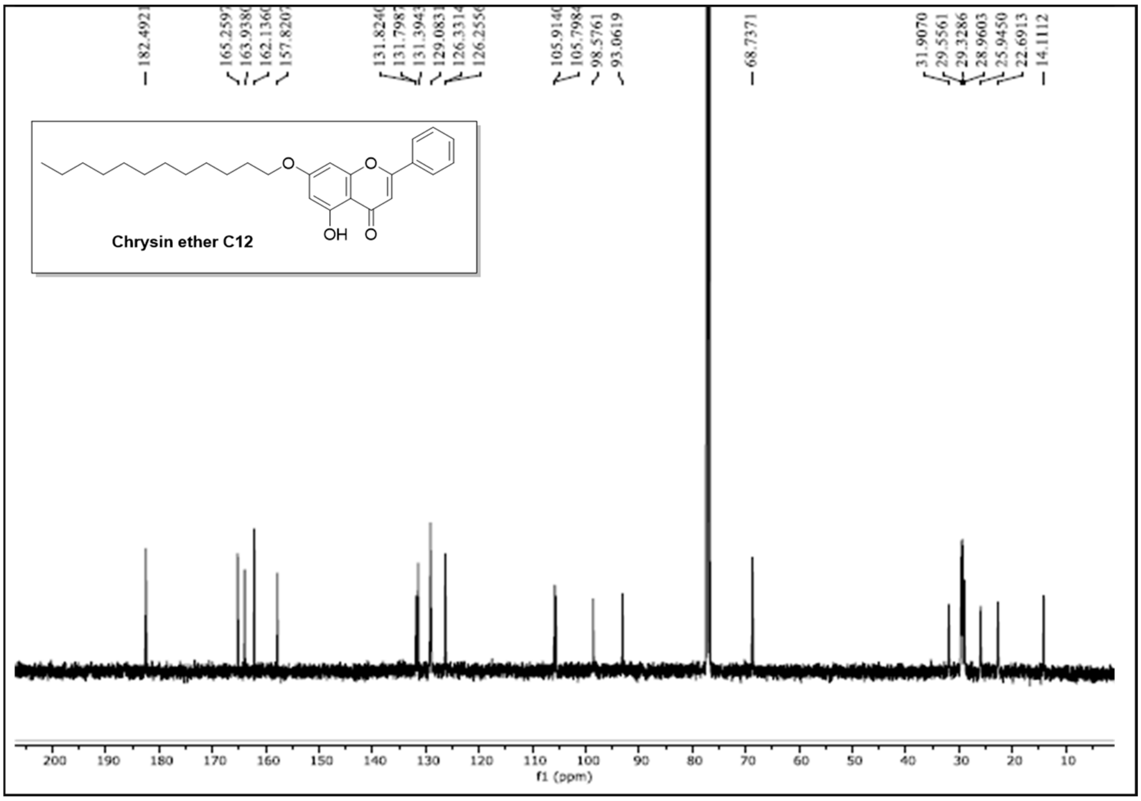

Supplementary Figure 10: ^{13}C NMR ($CDCl_3$, 500 MHz) spectrum of **Chrysin ether C12**.

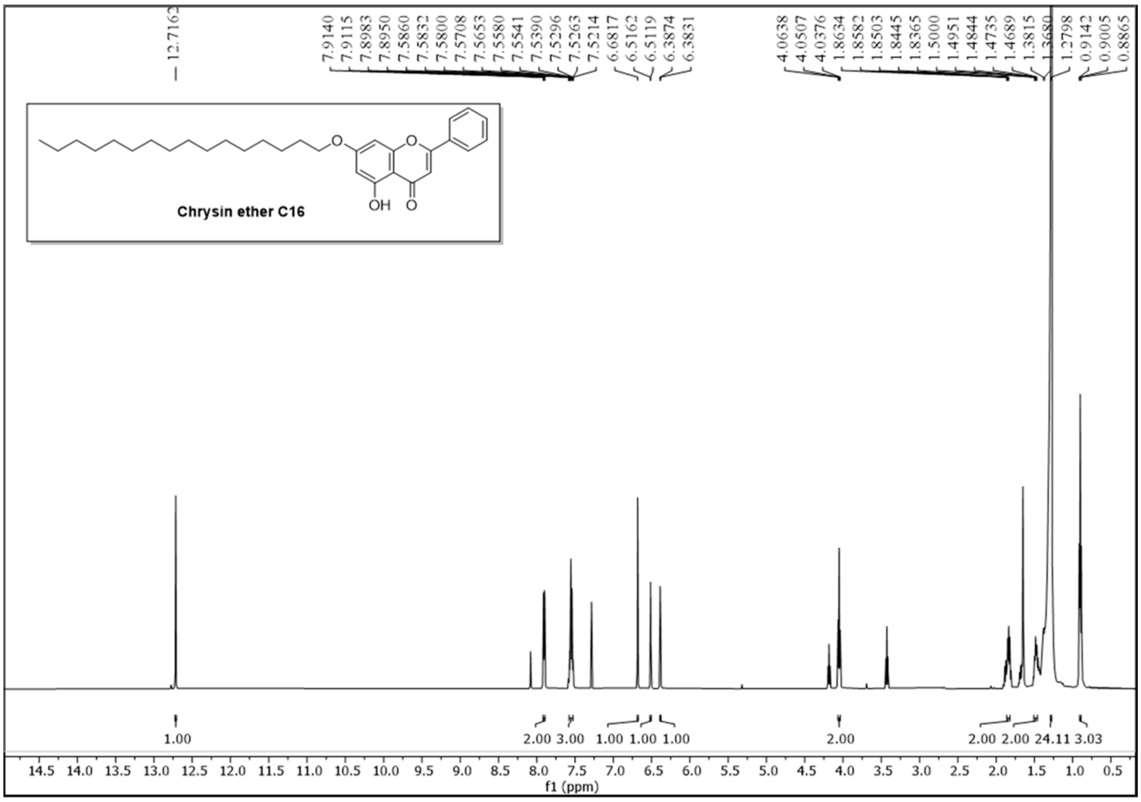

Supplementary Figure 11: ^1H NMR ($CDCl_3$, 500 MHz) spectrum of **Chrysin ether C16**.

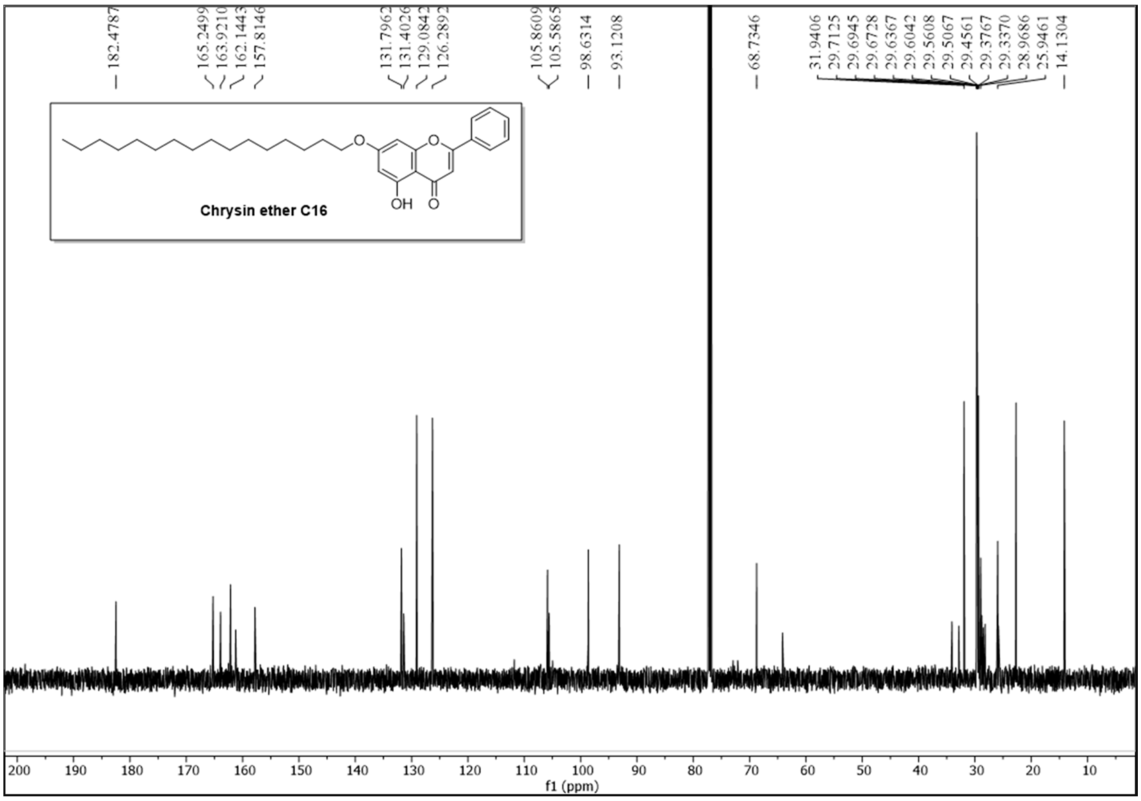

Supplementary Figure 12: ^{13}C NMR ($CDCl_3$, 500 MHz) spectrum of **Chrysin ether C16**.

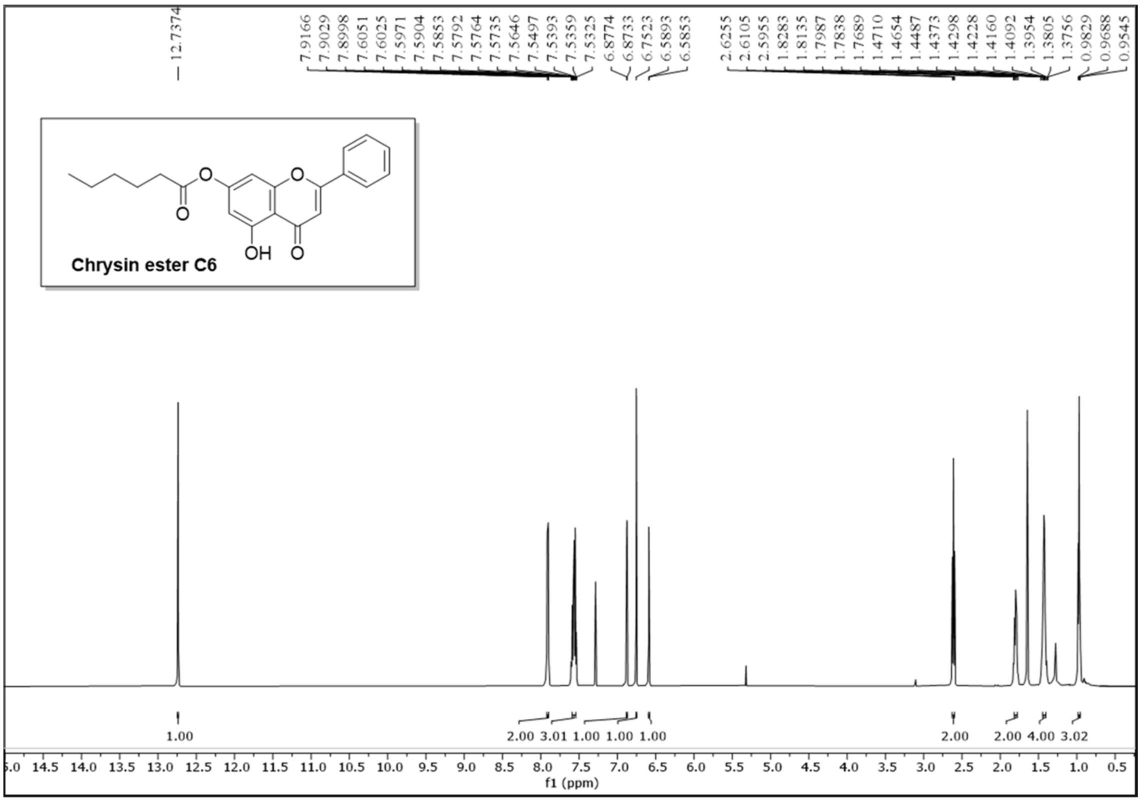

Supplementary Figure 13: ^{1}H NMR ($CDCl_3$, 500 MHz) spectrum of **Chrysin ester C6**.

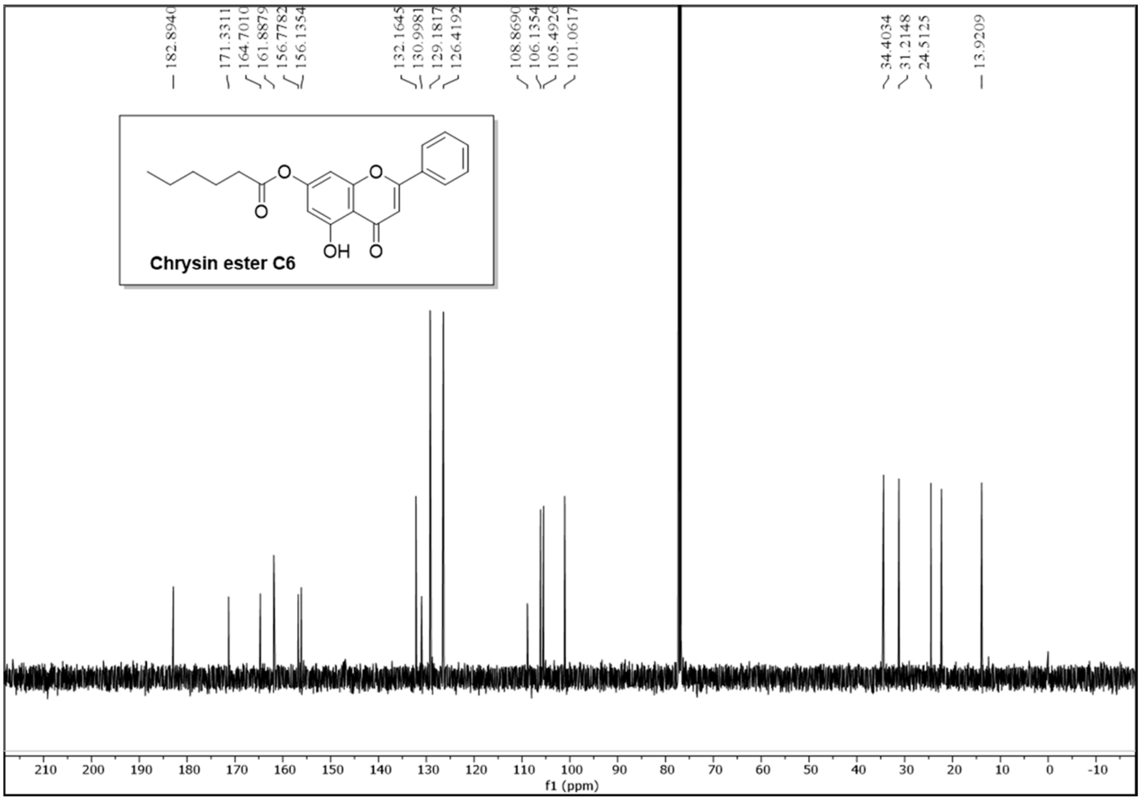

Supplementary Figure 14: ^{13}C NMR ($CDCl_3$, 500 MHz) spectrum of **Chrysin ester C6**.

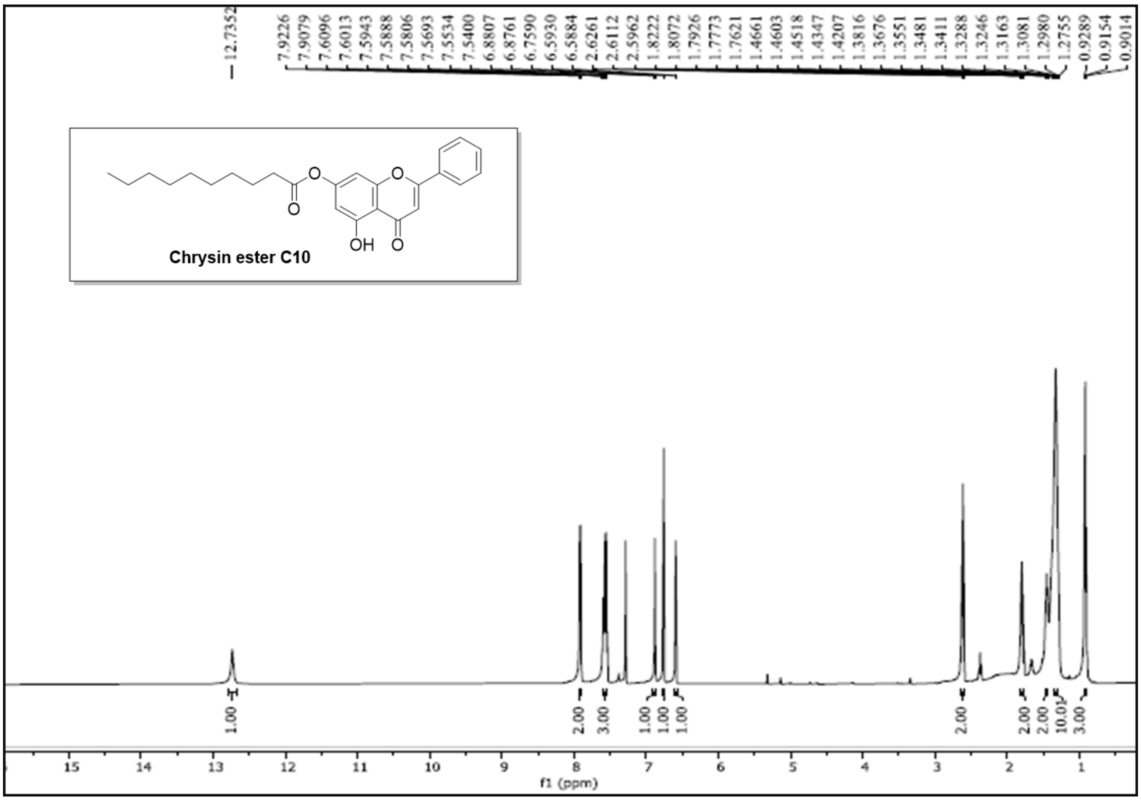

Supplementary Figure 15: ^{1}H NMR ($CDCl_3$, 500 MHz) spectrum of **Chrysin ester C10**.

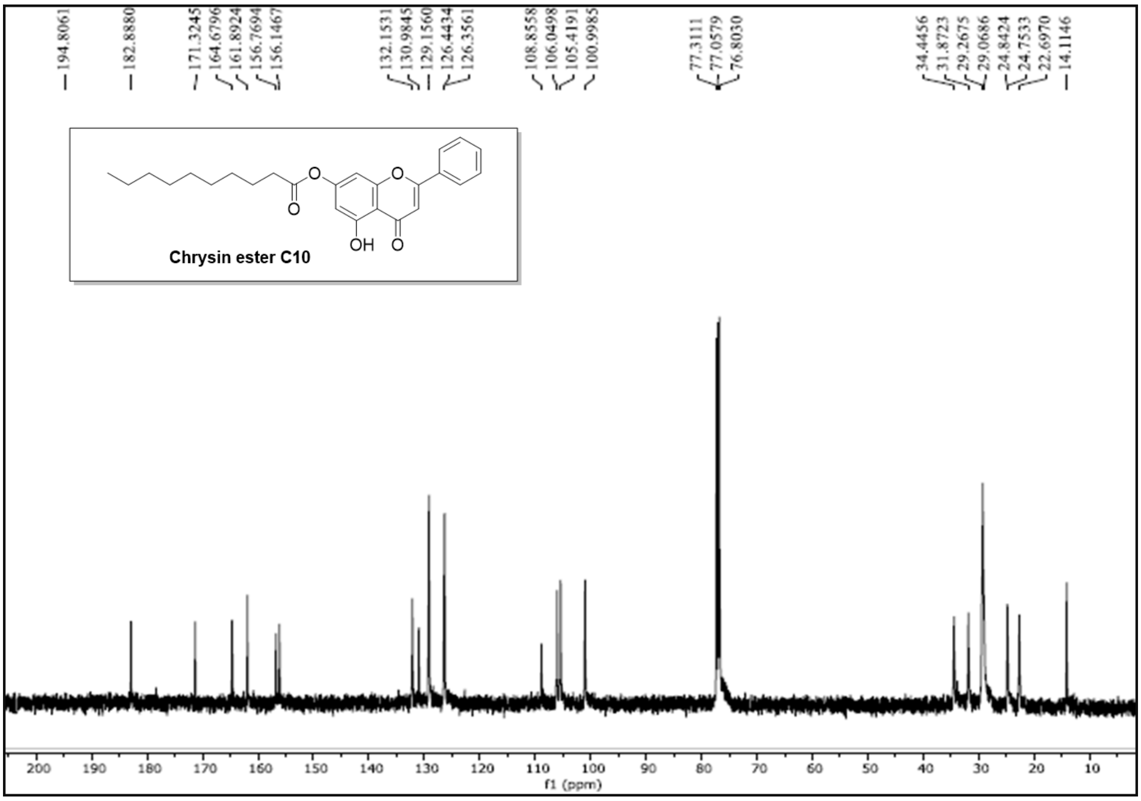

Supplementary Figure 16: ^{13}C NMR ($CDCl_3$, 500 MHz) spectrum of **Chrysin ester C10**.

References

1. Albuquerque de Oliveira Mendes, L.; Ponciano, C.S.; Depieri Cataneo, A.H.; Wowk, P.F.; Bordignon, J.; Silva, H.; Vieira de Almeida, M.; Ávila, E.P. The Anti-Zika Virus and Anti-Tumoral Activity of the Citrus Flavanone Lipophilic Naringenin-Based Compounds. *Chem. Biol. Interact.* **2020**, *331*, 109218, doi:10.1016/j.cbi.2020.109218.

2. Cheng, N.; Yi, W.-B.; Wang, Q.-Q.; Peng, S.-M.; Zou, X.-Q. Synthesis and α-Glucosidase Inhibitory Activity of Chrysin, Diosmetin, Apigenin, and Luteolin Derivatives. *Chinese Chem. Lett.* **2014**, *25*, 1094–1098, doi:10.1016/j.cclet.2014.05.021.

3. Zhu, Z.-Y.; Wang, W.-X.; Wang, Z.; Chen, L.-J.; Zhang, J.-Y.; Liu, X.; Wu, S.; Zhang, Y. Synthesis and Antitumor Activity Evaluation of Chrysin Derivatives. *Eur. J. Med. Chem.* **2014**, *75*, 297–300, doi:10.1016/j.ejmech.2013.12.044.

4. de Castro, P.P.; Rimulo, I.M.R.; de Almeida, A.M.; Diniz, R.; Amarante, G.W. Brønsted Acid-Catalyzed Epimerization-Free Preparation of Dual-Protected Amino Acid Derivatives. *ACS Omega* **2017**, *2*, 2967–2976, doi:10.1021/acsomega.7b00523.

5. de Castro, P.P.; Campos, D.L.; Pavan, F.R.; Amarante, G.W. Dual-protected Amino Acid Derivatives as New Antitubercular Agents. *Chem. Biol. Drug Des.* **2018**, *92*, 1576–1580, doi:10.1111/cbdd.13315.
